# Using machine learning to develop a five-item short form of the children’s depression inventory

**DOI:** 10.1186/s12889-024-18657-w

**Published:** 2024-04-23

**Authors:** Shumei Lin, Chengwei Wang, Xiuyu Jiang, Qian Zhang, Dan Luo, Jing Li, Junyi Li, Jiajun Xu

**Affiliations:** 1 College of Psychology, Sichuan Normal University, Chengdu, Sichuan China; 2 Mental Health Center, West China Hospital, Sichuan University, Chengdu, Sichuan China; 3 Department of Integrated Traditional and Western Medicine, West China Hospital, Sichuan University, Chengdu, Sichuan China; 4 Sichuan Key Laboratory of Psychology and Behavior of Discipline Inspection and Supervision, Sichuan Normal University, Chengdu, China

**Keywords:** Machine learning, CDI, Depression, Adolescents, Children

## Abstract

**Background:**

Many adolescents experience depression that often goes undetected and untreated. Identifying children and adolescents at a high risk of depression in a timely manner is an urgent concern. While the Children’s Depression Inventory (CDI) is widely utilized in China, it lacks a localized revision or simplified version. With its 27 items requiring professional administration, the original CDI proves to be a time-consuming method for predicting children and adolescents with high depression risk. Hence, this study aimed to develop a shortened version of the CDI to predict high depression risk, thereby enhancing the efficiency of prediction and intervention.

**Methods:**

Initially, backward elimination is conducted to identify various version of the short-form scales (e.g., three-item and five-item versions). Subsequently, the performance of five machine learning (ML) algorithms on these versions is evaluated using the area under the ROC curve (AUC) to determine the best algorithm. The chosen algorithm is then utilized to model the short-form scales, facilitating the identification of the optimal short-form scale based on predefined evaluation metrics. Following this, evaluation metrics are computed for all potential decision thresholds of the optimal short-form scale, and the threshold value is determined. Finally, the reliability and validity of the optimal short-form scale are assessed using a new sample.

**Results:**

The study identified a five-item short-form CDI with a decision threshold of 4 as the most appropriate scale considering all assessment indicators. The scale had 81.48% fewer items than the original version, indicating good predictive performance (AUC = 0.81, Accuracy = 0.83, Recall = 0.76, Precision = 0.71). Based on the test of 315 middle school students, the results showed that the five-item CDI had good measurement indexes (Cronbach’s alpha = 0.72, criterion-related validity = 0.77).

**Conclusions:**

This five-item short-form CDI is the first shortened and revised version of the CDI in China based on large local data samples.

## Background

The Report on National Mental Health Development in China (2019–2020) indicates that the prevalence of depression among Chinese teenagers in 2020 was 24.6% [1]. Liu and colleagues discovered that the prevalence of depression among Chinese secondary school students is remarkably higher than that of adults [2]. The detection rate of depression among primary school students in China is statistically significantly higher than in several countries, such as the United States, Greece, and Cyprus [3]. Additionally, as the population of China is vast and the resources for psychiatric health services are extremely limited, many young people could be living with undiagnosed depression. Most patients are first seen in non-psychiatric departments of the general hospital, resulting in a high misdiagnosis and false negative rate [4]. Researchers highlighted the need to conduct targeted screening of children aged 6–11 years with a high risk of depression and universal screening of adolescents aged 12–18 years to ensure early detection and treatment of depression [5]. However, despite being a widely-used depression prediction tool in China, the CDI has not yet been modified based on local samples, and previous studies have found cultural differences affecting its applicability in the Chinese context. Additionally, given the high prevalence of depression among Chinese teenagers and the limited resources available for psychiatric health services, there is a need for a fast and easy-to-use tool for depression prediction. Thus, the present study aimed to develop a localized and simplified depression measurement tool for Chinese children and adolescents.

### Development and application of the children’s depression inventory

Studies on depression in children in China have used the CDI as one of the most familiar measures with the broadest range of applications and low requirements for children’s reading levels. The CDI was developed by Kovacs from the adult version of Beck’s Depression Inventory to measure depression in children and adolescents aged 7 to 17 years [6]. It is a 27-item self-report questionnaire, with each item containing three options for describing severity, each scored from 0 to 2. The scale’s total score is 54, with higher scores indicating more severe depression and a threshold of 20 for depression screening [7]. The CDI is widely adopted as one of the most feasible tools to assess depressive symptoms in children and has been demonstrated to have excellent measurements [8, 9]. The 10-item short version Children’s Depression Inventory-Short Version (CDI-S) was originally developed as a rapid version of the original CDI, which was subsequently extended with the 28-item revised version of the full-length questionnaire (CDI-II), and the 12-item short version (CDI-II-S). Comparatively, none of these three versions have been widely accepted in research and have thus only been used in a few studies [10].

On platforms such as China National Knowledge Infrastructure and China Science and Technology Journal Database, no studies were found that have adapted the CDI based on a sample of Chinese children. Most of the research on depression in Chinese adolescents and children used the Chinese translation of the CDI. Nevertheless, previous studies have identified some problems with the reliability of the CDI subscales, which have been attributed to cultural differences or translation accuracies. For example, studies have found that the internal consistency coefficients of the ineffectiveness, negative self-esteem, and interpersonal problems subscales are below 0.6 [8]. Chinese scholars have not yet localized and revised the CDI using samples of Chinese children, despite its structural instability and poor subscale measures. Therefore, the study aimed to optimize the CDI using a sample of 20,695 Chinese children to provide a reference for the subsequent localization and simplification of the CDI.

### Simplifying CDI with machine learning

The mental health screening of children and adolescents has received increasing attention. Scales are widely used for screening because of their ease of administration and the speed with which results can be obtained [11]. However, there are limitations to using scales in clinical practice. For example, individuals in the physical examination scenario are less compliant and struggle to complete scales that are overloaded with items [12]. Prolonged test administration is also likely to contribute to fatigue, which will affect the reliability of the results [13]. In addition, children and adolescents, still at varying self-control and cognitive development stages, have difficulty completing large scales [14]. As such, lengthy scales are not ideal to use where the target group for screening is children with depression, who often exhibit poor concentration and irritability [15]. The scale to be used should thus be simplified as much as possible to suit the target group’s developmental stage and cognitive characteristics, to ensure accurate screening. Although short versions of the CDI have been published, with 10 and 12 items, these still require up to 10 min to complete. To address the lack of research on shortened versions of the CDI in China [9, 16, 17], the study will develop screening scales with as few items as possible based on local samples. The aim is to help improve the efficiency of clinical screening and diagnosis and reduce under-diagnosis and misdiagnosis of children and adolescents with depression in China.

Existing scale simplification studies have tended to use the factor analysis methodology. However, the structural dimensions of the CDI are unstable and consistently exist in multiple interpretative versions [7, 18, 19]. Thus, a factor analysis approach based on the original structure would be unsuitable. When using confirmatory factor analysis, researchers must ensure that at least two to three items are retained under each dimension to identify problems and test for effectiveness, thus limiting the effectiveness of simplifying the scale in practice [20]. An emerging solution lies in ML, which automatically improves algorithms and captures potential patterns in data by learning from existing data, followed by analysis and prediction of unknown data [21, 22]. Without the need to consider the dimensions of the scale during the analysis, ML can overcome the dimensional limitations of the factor analysis method, thus further reducing the number of scale items. Consequently, using ML to revise and simplify the CDI will overcome the limitations of traditional factor analysis methods as well as represent and reflect the characteristics of the local sample data.

Numerous scholars have attempted to apply ML algorithms to optimize psychiatric scales. Wall et al. used a ML algorithm to simplify the Autism Diagnostic Interview-Revised from 93 to 7 items, and they obtained a near perfect accuracy based on a validation of two different datasets [23]. Sun et al. employed six ML algorithms to reduce the items on the Minnesota Multiphasic Personality Inventory by approximately 40%, achieving 85% of the sensitivity and specificity of the original test [24]. Meanwhile, other studies have used ML algorithms to screen different social groups for mental illness based on cross-sectional clinical data with positive results [25]. It was found that more than 190 studies have applied ML to the detection and diagnosis of psychiatric disorders, including but not limited to depression, schizophrenia, and Alzheimer’s disease [26]. The related findings suggest that ML could effectively identify potential predictors of postpartum depression and improve its early detection rate [27]. Some scholars point out that ML can help mental health practitioners define mental illness more objectively, contribute to the early identification of illness and effective intervention, and promote the development of psychiatric and psychological disciplines [28, 29]. In addition, the application of ML in liquid biopsy of cancer and radiotherapy of tumors has also achieved milestones [30–32]. Thus, ML techniques have been used extensively for scale revision and the screening and treatment of various diseases, yielding promising results. The use of ML to revise the CDI would maximize the simplicity of the scale length while ensuring the validity and accuracy of the screening results. Therefore, the ML method was used to simplify the CDI in this study.

In summary, the present study will develop a short-form CDI using ML, which may enhance the efficiency of predicting high depression risk in children and adolescents. The short-form CDI can facilitate large-scale and rapid screening, thereby ensuring timely attention and intervention for children and adolescents at high risk of depression.

## Methods

### Samples

The present study contains two phases: a). the simplification phase, and b). the validation phase. The data for the simplification phase was derived from a post-earthquake child mental health survey conducted from May to July 2009 in Qingchuan County, Guangyuan City, Sichuan Province. During that period, the second corresponding author of this study, in the role of a psychological assistant and volunteer, conducted a large-scale psychological survey alongside their research team at local primary and secondary schools. The survey aimed to assess the depression (using the CDI scale) and anxiety levels among 21,652 children aged 7 to 15 years face-to-face at school (without the presence of a guardian); ultimately, 20,749 children completed the scale. The dataset used in this study was anonymized and preprocessed based on the raw data (available at https://osf.io/a68ft). It includes clinicians’ evaluations of depression for all participants. After excluding missing and invalid data (item scores other than 0, 1, or 2), 20,675 valid datasets were retained. Thus, the data for the simplification phase include details of each item in the CDI for 20,675 participants. The mean age of the sample was 11.66 ± 2.28 years; 49.84% (10,305) were male. Individuals with CDI scores exceeding 20 was screened as a risk group. Subsequently, professional volunteers from various parts of the country, including psychology teachers, psychiatrists, graduate students in psychology or psychiatry, conducted structured interviews with these at-risk individuals to confirm the presence of depressive symptoms. A total of 6,436 children were identified as being at risk of depression. 70% of the data were randomly divided into training samples for training the ML model; the remaining 30% were used as test samples to test the predictive performance of the ML model.

In the validation phase, we tested the short-form CDI scale in November 2022, Sichuan Province secondary school students. Three hundred and eighty-nine questionnaires were collected, excluding 68 that were not answered carefully and 6 that were incomplete. Finally, we obtained 315 valid questionnaires with a valid recall rate of 80.98%. The mean age of the 315 participants was 14.5 years (SD = 1.47), ranging from 12 to 18 years old, and 165 (52.38%) were women. Their grade distribution ranged from junior to senior, with 66.98% junior high school students (*n* = 211, 51.66% female) and 33.02% high school students (*n* = 104, 50.96% female).

### Analysis methods

#### Procedures of simplifying CDI

**Fig. 1:**
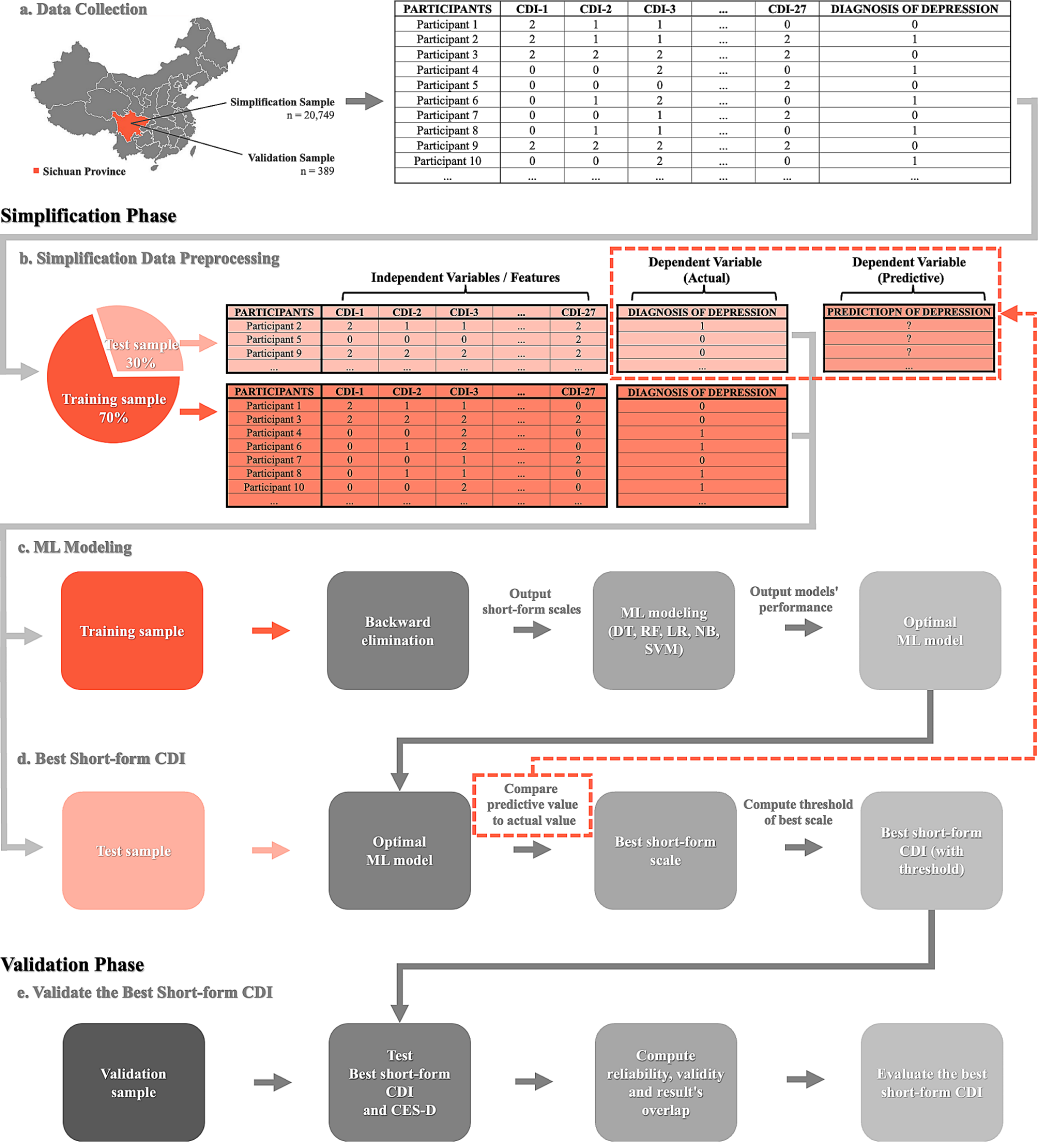
Procedures of simplifying CDI

The procedure of simplifying CDI includes the simplification phase and the validation phase (Fig. 1). The dataset consists of a simplification sample and a validation sample: the former is utilized for simplifying the full-version CDI, while the latter is collected post-simplification and employed to validate the optimal short-form CDI.

In the simplification phase, raw simplification data necessitates preprocessing prior to modeling. 70% of the simplification data were randomly assigned to the training set for ML model training, with the remaining 30% serving as the test set to assess the ML model’s predictive performance. The 27 items in CDI detail independent variables (or features), while the corresponding depression diagnosis constitutes the actual dependent variable (i.e., clinicians’ evaluations of depression).

The initial step involves utilizing backward elimination in training set to compute the contribution of each item and generate a list based on these contribution values, ranked from highest to lowest (Fig. 1c). Subsequently, based on this list, the items for each short-form scale are determined. For instance, the top three variables in the backward elimination contribution list (e.g., CDI 7, CDI 10, and CDI 20) are designated as the items for the three-item short-form scale. The next step entails fitting ML models for each short-form scales (e.g., fitting five ML models for both three-item and four-item scales) and confirm the optimal model (e.g., naïve Bayes) according to the AUC. Following this, the evaluation metrics (e.g., accuracy, recall, and AUC) of the optimal model (e.g., naïve Bayes) for each short-form scale are compared to ascertain the most effective one. Once the best short-form scale is determined (e.g., the five-item scale), a confusion matrix is generated using the predictive and actual results of the best short-form scale (Fig. 1d). Subsequently, the optimal decision threshold is established based on the confusion matrix at various potential thresholds. Here we get the best short-form CDI with a threshold. Evaluation metrics encompass accuracy, recall, precision, F1 score, and AUC, each serving distinct evaluation purposes. In this study, the confusion matrix is a 2 × 2 table employed in machine learning to assess classification model performance, utilizing four crucial metrics: True Positives (TP), True Negatives (TN), False Positives (FP), and False Negatives (FN). It aids in quantifying model performance metrics like accuracy, recall, and precision.

Moving to the validation phase (Fig. 1e), the reliability and validity of the best short-form CDI are further tested in a new independent sample.

#### Machine learning

In the ML model employed in this study, scores on each item serve as independent variables (features), while depression classification, determined through structured interviews, serves as the dependent variable. The trained ML model predicts whether a participant is depressed based on their scores on each item. It is crucial to note that this classification is predictive and not equivalent to a clinician’s diagnosis. Depression prediction is treated as a dichotomous variable (positive or negative), thus this study employed classification algorithms commonly used in ML, including decision tree (DT), logistic regression (LR), naïve Bayes (NB), random forests (RF), and support vector machines (SVM), totaling five ML algorithms [33, 34].

Prior to modeling, data is typically divided into two subsets: a training set, consisting of approximately 70% of the original data, and a test set, containing the remaining 30%. Different models or algorithms are then trained on the training set, producing trained models. These models are utilized to predict outcomes, such as identifying high or low depression risk, by inputting independent variables or features from the test set. Model fitness is evaluated by comparing these predictive values with actual results.

Common evaluation metrics for classification models include accuracy, recall, precision, F1 score, and AUC. In interpreting the evaluation metrics, “P” and “N” referred to the depressed and non-depressed samples, respectively, as determined by the full version of the CDI. AUC reflects the capacity of the algorithm to discriminate between depressed and non-depressed samples. AUC = 0.5 indicates that the depressed sample could not be distinguished using the model, and AUC = 1 indicates that the model could distinguish the depressed sample with 100% success. An AUC closer to 1 indicate better model performance [35]. Accuracy is the ratio of samples correctly classified by the model to the total number of samples; the value ranges between 0 and 1, and the larger the value, the better the model performance. Recall is the ratio of depressed samples correctly classified by the model to the total number of P-samples, with larger values indicating that the model identified more depressed samples and values ranging between 0 and 1 [36]. Precision is the ratio of depressed samples correctly classified by the model to the total number of depressed samples classified by the model, reflecting the extent to which the depression predictions made by the model are reliable [36]. F1 score is the harmonic mean of recall and precision, and its value ranges between 0 and 1. A larger F1 score indicates better model performance [34].

## Results

### Simplification of CDI

In order to reduce the number of items on the short-form to 30% or more of the full version of the CDI, the analysis focused on short-form scales containing 1 to 9 items. The optimal items for different lengths of short-form scales were determined using the backward elimination algorithm (Table 1).

**Table 1:** The best Item(s) for different short-form scales

Items	CDI 3	CDI 7	CDI 10	CDI 11	CDI 12	CDI 16	CDI 17	CDI 20	CDI 21
1								*	
2		*						*	
3		*	*					*	
4		*	*					*	*
5		*	*				*	*	*
6	*	*	*				*	*	*
7	*	*	*	*			*	*	*
8	*	*	*	*		*	*	*	*
9	*	*	*	*	*	*	*	*	*

Note: CDI-X indicates the serial number of the item in the CDI.

Using ML algorithms to model the short-form scales, all models had AUCs above the 0.5 (Table 2), with a maximum value of 0.87 and a minimum value of 0.61. Because AUCs for short-form scales with fewer than five items were consistently lower than 0.8, subsequent analyses focused on five to nine-item scales to ensure that short-form scales could be implemented more effectively. In addition, it was found that the AUCs of the DT and LR were consistently lower than the other three algorithms by comparing the AUCs of the five algorithms on the five to nine-item scales.

**Table 2:** AUCs for ML models of short-form scales

Items	DT	LR	NB	RF	SVM
1	0.61	0.61	0.61	0.61	0.61
2	0.72	0.64	0.76	0.72	0.64
3	0.78	0.74	0.75	0.74	0.76
4	0.77	0.74	0.78	0.79	0.77
5	0.77	0.77	0.81	0.80	0.81
6	0.77	0.80	0.82	0.82	0.82
7	0.77	0.80	0.83	0.83	0.84
8	0.77	0.82	0.84	0.84	0.84
9	0.77	0.83	0.85	0.87	0.86

**Fig. 2:**
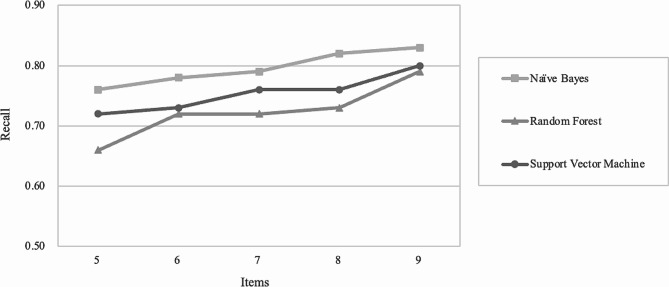
Recall for ML models of short-form scales

Given the above, the recalls of the NB, RF, and SVM on five to nine-item scales were further analyzed. The recall is the proportion of samples classified as depressed by the full version of the CDI correctly identified by the model, with larger values indicating that the model is less likely to miss depressed samples. Since the recalls of the NB were consistently higher than the other two algorithms (Fig. 2), the NB model’s evaluation metrics were adopted to estimate the performance of the five to nine-item scales.

Comparing the accuracy, recall, precision, and AUC of the NB on a five to nine-item scale (Table 3) revealed that the model’s predictive performance improved weakly as the number of items increased. Comparing the five-item scale with the 9-item scale, the differences in accuracy, recall, precision, and AUC are 0.03, 0.07, 0.03, and 0.04, respectively, but the difference in the number of items is nearly doubled. Given the low marginal benefit with increasing items, the five-item scale is chosen as a short-form scale for efficient screening of high depression risk groups.

**Table 3:** Analysis of the CDI short-form scale using the NB

Items	Accuracy	Recall	Precision	AUC
5	0.83	0.76	0.71	0.81
6	0.84	0.78	0.72	0.82
7	0.85	0.79	0.74	0.83
8	0.85	0.82	0.74	0.84
9	0.86	0.83	0.74	0.85

Developing the short-form scale aims to help staff could screen for people at high risk of depression based on the scale without needing a background in psychology or clinical medicine or the use of computational tools. Therefore, an optimal decision threshold for the short-form scales was needed to help staff quickly classify respondents. Ten confusion matrices were generated based on different thresholds (scores from 1 to 10), then ten sets of AUCs, accuracies, recalls, and precisions were obtained (Table 4). When the thresholds were 3 and 4, the AUCs and accuracies were more significant than 0.8, and the predictive performance of the scales was satisfactory. However, there is a slight complication between recall and precision. As the threshold value is raised or lowered, there is a reciprocal relationship between recall and precision. A higher Recall indicates a lower rate of missed alarms, and a higher precision indicates a lower rate of false alarms. For the scale, increasing the threshold will decrease the false alarm rate but also increase the missed alarm rate; decreasing the threshold will decrease the missed alarm rate but also increase the false alarm rate.

**Table 4:** Evaluation indicators corresponding to different thresholds of the five-item scale

Threshold	AUC	Accuracy	Recall	Precision
1	0.65	0.52	1.00	0.39
2	0.77	0.69	0.97	0.50
3	0.83	0.81	0.89	0.64
4	0.81	0.85	0.72	0.78
5	0.73	0.82	0.49	0.88
6	0.64	0.77	0.28	0.95
7	0.56	0.73	0.13	0.98
8	0.52	0.70	0.05	0.99
9	0.51	0.69	0.02	1.00
10	0.50	0.69	0.00	1.00

**Fig. 3:**
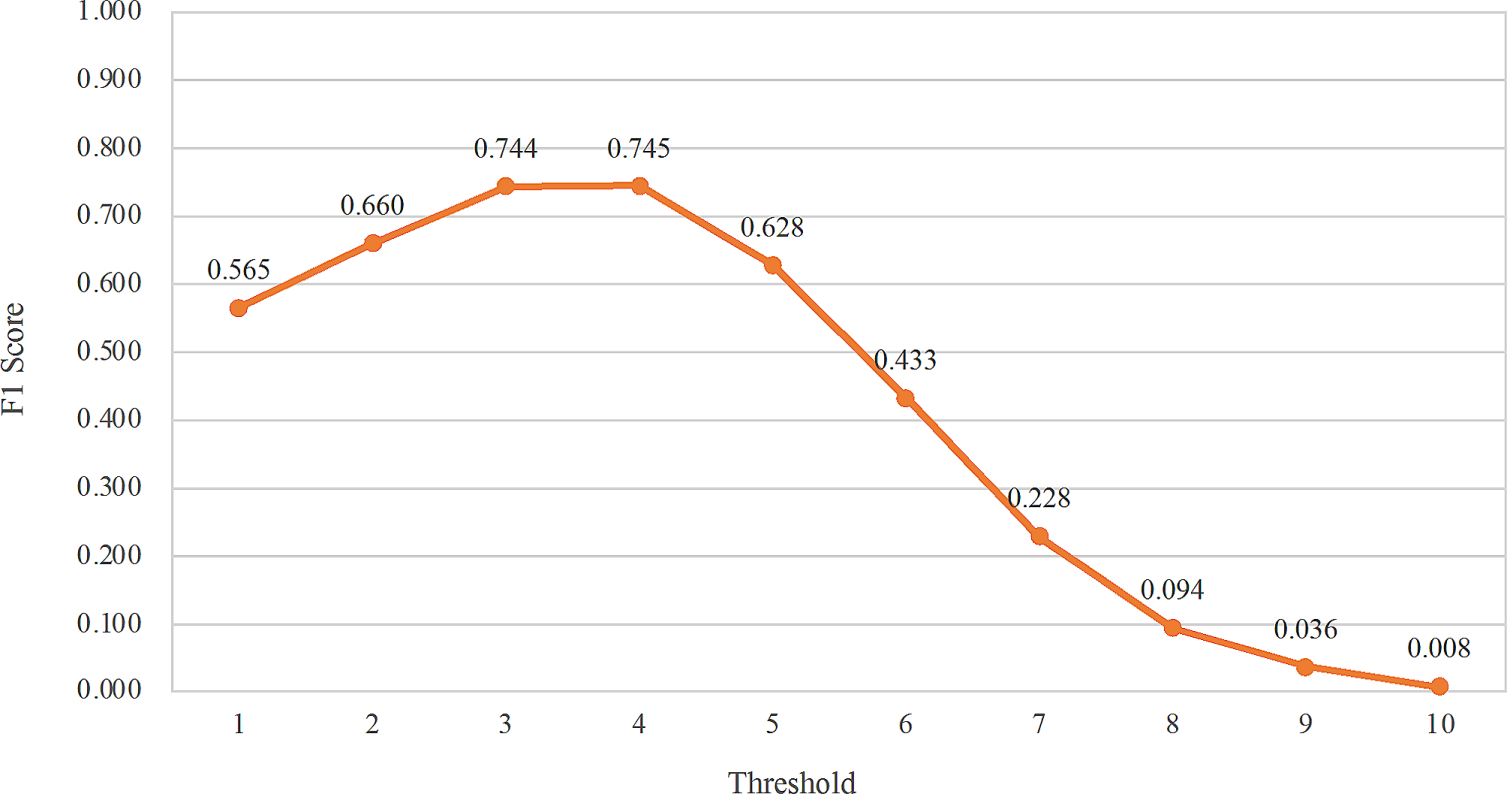
F1 scores for short-form CDI with different thresholds

The assumption is that clinicians neither want too low a threshold to cause more false positives nor too high a threshold to cause too many missed detections. Therefore, to better weigh recall and precision, their harmonic mean, the F1 score, was used as a measure of the predictive performance of the five-item short-form scales in different threshold situations. As the decision threshold increases, the F1 score becomes larger and larger, and the F1 score is most significant when the threshold is 4, after which the F1 score becomes smaller and smaller as the threshold increases (Fig. 3). Therefore, the optimal decision threshold for the five-item scale was finally determined to be 4.

### Validation of the five-item CDI

After obtaining the optimal five-item CDI, it was retested in a sample of middle school students. SPSS 22.0 was used for analysis to test the internal consistency reliability, split-half reliability, criterion-related validity, and construct validity of the five-item CDI. The results showed that the internal consistency reliability of the five-item CDI was 0.72 (*p* < 0.01). Subsequently, unequal Spearman-Brown coefficients of 0.73 (*p* < 0.01) and Guttman coefficient of 0.71 (*p* < 0.01) were obtained by the odd-even split-half method, indicating good reliability of the scale.

The Center for Epidemiologic Studies Depression Scale (CES-D) was chosen as the calibration scale for this study due to its wide usage and good reliability and validity in depression research among Chinese children and adolescents [37, 38]. The CES-D consists of 20 items and contains four dimensions (somatic symptoms, depressed mood, positive mood, and interpersonal problems), a score of 28 is generally used as the cut-off score for depression screening. Similar to the CDI, the CES-D requires subjects to self-report their depression symptoms over the past week, where higher total item scores indicate greater levels of depression [37, 38]. The scale has been widely used to assess depression levels among Chinese adolescents aged 11 to 19 years, demonstrating good reliability and validity [39–41]. In the present study, the internal consistency reliability of CES-D was 0.91 (*p* < 0.01), and the criterion-related validity of CES-D and five-item CDI was 0.77 (*p* < 0.01). The correlation coefficients between the five total CDI scores and each item score ranged from 0.52 to 0.79 (*p* < 0.01) (Table 5), and were significantly and positively correlated with the total CES-D score as well as with the four sub-dimension scores (Table 6). The overlap between the results of the five-item scale (with a cut-off score of 4) and CES-D (with a cut-off score of 28) is 93.97%.

**Table 5:** Correlation matrix for each item and total of the five-item short-form CDI

	CDI 7	CDI 10	CDI 17	CDI 20	CDI 21	Total
CDI 7	1.00					
CDI 10	0.24^**^	1.00				
CDI 17	0.42^**^	0.25^**^	1.00			
CDI 20	0.49^**^	0.34^**^	0.46^**^	1.00		
CDI 21	0.34^**^	0.16^**^	0.30^**^	0.43^**^	1.00	
Total	0.72^**^	0.52^**^	0.75^**^	0.79^**^	0.65^**^	1.00

Note: ^**^P < 0.01(Two-tailed test)

**Table 6:** Correlation matrix for the five-item short-form CDI and each dimension of the CES-D

	Depressed affect	Positive affect	Somatic and retarded activity	Interpersonal	CES-D Total
CDI-5 Total	0.72^**^	0.57^**^	0.67^**^	0.53**	0.77^**^

Note: ^**^P < 0.01(Two-tailed test)

The Kaiser-Meyer-Olkin Test (KMO) is a method that evaluates the data suited for factor analysis. Generally, KMO > 0.6 is acceptable. Statistical results showed that KMO = 0.78 > 0.6 and Bartlett’s spherical test *p* < 0.005, indicating that the five-item scales were suitable for factor analysis. Later, exploratory factor analysis was performed on the five-item CDI using the principal component analysis. One common factor was extracted with the criterion of eigenroot > 1, and the cumulative variance contribution rate was 48.096%. The factor loads of the five items on the common factor from high to low were 0.81, 0.74, 0.71, 0.64 and 0.52, which were all greater than 0.5. In summary, the five short-form CDI has an acceptable structural validity.

## Discussion

The study employs ML techniques to develop a five-item short-form CDI based on a large sample of 20,675 Chinese children. After considering all the indicators, we recommend the five-item short-form scale as the optimal choice, with an accuracy of 0.85, recall of 0.72, precision of 0.78, and AUC of 0.81. Compared to the original scale, the optimal short-form scale demonstrates a performance range of 72–85%, with a reduction in the number of items by 81.48%. In one study validating the ten-item CDI (with an optimal threshold of 3), the AUC and recall were found to be 0.88 and 0.93, respectively. Although the AUC and recall of the five-item short-form CDI (with the same threshold of 3) showed a reduction of about 5% and 4%, respectively, compared to the ten-item CDI’s performance, the number of items decreased by 50% [42]. Another study examining the twelve-item CDI revealed internal consistency reliability and AUC values of 0.66 and 0.75, respectively. Interestingly, the five-item CDI demonstrated higher values (0.72 and 0.81) and a reduction in item count by 58.33% [8].

The retest results showed that the five-item short-form CDI’s overall reliability and split-half reliability were above 0.65, with good reliability. The correlation between the short-form CDI total score and the CES-D and its dimensions ranged from 0.53 to 0.77. All the above results indicate that the five-item CDI have good reliability. In addition, the five-item CDI (with a cut-off score of 4) had a 94% overlap in outcomes with the full version of the CES-D, suggesting that this short version of the scale is effective in assessing depression risk in adolescents. The study aims to develop an efficient tool for predicting individuals at high risk of depression. This tool will help children and adolescents in receiving timely attention and intervention from professionals. It allows for individual assessment in just one minute, thereby reducing costs associated with group and community assessments.

The five-item short scale would be helpful for large-scale screening of depression and easy to use in assessing individuals who have the insufficient cognitive ability or cannot complete the full version of the scale owing to illness. Our five-item scale is also the first short version of the CDI revised based on a large sample of local data in China. It will be more suitable for Chinese children and adolescents and provide some reference for the localization revision and simplification of the CDI.

By comparison, it was found that the five-item CDI (Cronbach’s alpha = 0.72, split-half coefficient = 0.73) had a slightly lower internal consistency coefficient and split-half coefficient than the original CDI (Cronbach’s alpha > 0.82, split-half coefficient = 0.82), but the number of questions was only 18.51% of the original CDI [7, 19]. Previous studies have shown that the internal consistency reliability > 0.70 is acceptable [43]. Studies of the application of the CDI in Chinese groups have found that item 26 of the original CDI correlates with the total scale score below 0.15, and item 15 has an item loading of only 0.11 on the common factor [7, 19]. The five-item CDI did not include these two items, supporting the previous research conclusions. Although the five-item CDI does not contains all the five dimensions of the original CDI, it outperforms the CDI-II-S (Cronbach’s alpha = 0.66, AUC = 0.75, criterion-related validity = 0.37) and the CDI-S (criterion-related validity = 0.60 ~ 0.72) on all measures [10, 44]. Compared to other child depression assessment tools, the length of the five-item CDI was only 45.45% of the Kutcher Adolescent Depression Scale-11 (KADS-11) and 27.78% of the Depression Self-rating Scale for Children (DSRSC). However, the criterion-related validity and AUC of the five-item CDI were higher than those of the KADS-11 (criterion-related validity = 0.74, AUC = 0.7 ~ 0.9). The five-item CDI’s split-half reliability and internal consistency coefficients were comparable to the DSRSC (Cronbach’s alpha = 0.73, split-half reliability = 0.72). In summary, the five-item CDI shows excellent measurement metrics with the significant advantage of simplicity, no matter which perspective is considered.

The study also has some limitations. First, large sample data from China was used in the simplification stage of the scale, but the sample consisted of children affected by the Wenchuan Earthquake. Moreover, owing to the geographical limitation of the survey, most of the children were Han Chinese in rural areas. The sample in the simplification phase included children aged only 7–15 years; meanwhile, the CDI included children aged 15–17 years as well. Although the presence of 12–18 years of children in the retest stage to make up for this defect to some extent, whether the four-item short version CDI would be suitable for other child groups would need further verification. In the future, the research should further analyze the differentiated characteristics of depressed people at different ages and how to deal with the understanding of CDI by younger children. At the same time, because ML models will inevitably be limited by the data quality used to develop them, future researchers should strengthen their collaboration with clinicians. Having clinicians provide the data set used to train the model and their feedback on using the short version of the scale will maximize the usefulness of ML. Second, our five-item scale only contained the items of the subscales of Anhedonia and Negative Self Esteem in the original model of CDI. It has no items on the subscales of Negative Mood, Ineffectiveness, and Interpersonal Problems. The reason for this result may be that the samples used in this study and the original study for constructing the CDI scale are significantly different, that is, the samples in this study are from rural areas in western China, so the two populations may have different manifestations of depressive symptoms. Furthermore, a study on the utilization of the CDI in Chinese samples revealed that the factor structure of the CDI in Chinese populations differs from the original version, necessitating adjustments to the Interpersonal Problems subscale to align with cultural nuances [45]. The ten-item CDI in [45] is also a single-dimension scale. This study achieved good calibration and constructed validity of the five-item short-form scale. However, in practical clinical application, the short version of the scale can only serve as a reference for overall assessment of individuals’ depression status, without providing guidance on intervention direction or specific dimensions. Third, after obtaining the five-item short-form scale, reliability and validity tests were conducted in a small sample of Chinese children only. Future studies may include additional samples nationwide for testing and comparison with other depression measurement tools to validate and supplement the applicability of this five-item CDI.

## Conclusion

This study developed a five-item short-form CDI based on the original CDI using ML techniques. The five-item short-form CDI comprises CDI 7, CDI 10, CDI 17, CDI 20, and CDI 21 in the original CDI. Furthermore, the results of validation showed that the five-item CDI exhibits satisfactory reliability and validity. It can be used to predict high depression risk among children and adolescents in China, thereby enhancing the efficiency of identifying individuals warranting increased attention from professionals. While this study suggests a decision threshold of 4 for the five-item CDI, additional relevant parameters are also provided within the text to assist professionals in making the appropriate judgement tailored to the specific circumstances.

## Data Availability

This study was preregistered on OSF registries. The datasets used and/or analyzed during the current study are available online (https://osf.io/a68ft).

## Abbreviations

AUC: The area under the ROC curve
CDI: The Children’s Depression Inventory
CDI-S: The Children’s Depression Inventory-Short Version
CDI-II: the 28-item revised version of the full-length CDI questionnaire
CDI-II-S: the 12-item short version of CDI-II
CES-D: The Center for Epidemiologic Studies Depression Scale
DSRSC: The Depression Self-rating Scale for Children
DT: Decision Trees
KADS-11: Kutcher Adolescent Depression Scale-11
KMO: Kaiser-Meyer-Olkin Test
ML: Machine Learning
LR: Logistic Regression
NB: Naïve Bayes
RF: Random Forests
SVM: Support Vector Machine
TP: True Positives
TN: True Negatives
FP: False Positives
FN: False Negatives
